# Supplementary material for: A Critical View of the Application of the APEX Software (Aqueous Photochemistry of Environmentally-Occurring Xenobiotics) to Predict Photoreaction Kinetics in Surface Freshwaters
Source: Molecules. 2019 Dec 18;25(1):9. doi: 10.3390/molecules25010009 (PMC7017383; doi:10.3390/molecules25010009)
Supplement: Supplementary file 1 [file molecules-25-00009-s001.zip › 3.2.4_gcc-4.4.0.zip!/3.2.4_gcc-4.4.0/3.2.4_gcc-4.4.0/doc/octave/html/interpreter/Input-Conversion-Syntax.html]

Input Conversion Syntax - Untitled


Next: Table of Input Conversions,
Previous: Formatted Input,
Up: C-Style I/O Functions


---

#### 14.2.12 Input Conversion Syntax

A `scanf` template string is a string that contains ordinary
multibyte characters interspersed with conversion specifications that
start with %.

Any whitespace character in the template causes any number of whitespace
characters in the input stream to be read and discarded. The whitespace
characters that are matched need not be exactly the same whitespace
characters that appear in the template string. For example, write
 ,  in the template to recognize a comma with optional whitespace
before and after.

Other characters in the template string that are not part of conversion
specifications must match characters in the input stream exactly; if
this is not the case, a matching failure occurs.

The conversion specifications in a `scanf` template string
have the general form:

```
     % flags width type conversion
```

In more detail, an input conversion specification consists of an initial
% character followed in sequence by:

- An optional flag character \*, which says to ignore the text
  read for this specification. When `scanf` finds a conversion
  specification that uses this flag, it reads input as directed by the
  rest of the conversion specification, but it discards this input, does
  not return any value, and does not increment the count of
  successful assignments.
  - An optional decimal integer that specifies the maximum field
    width. Reading of characters from the input stream stops either when
    this maximum is reached or when a non-matching character is found,
    whichever happens first. Most conversions discard initial whitespace
    characters, and these discarded characters don't count towards the
    maximum field width. Conversions that do not discard initial whitespace
    are explicitly documented.
    - An optional type modifier character. This character is ignored by
      Octave's `scanf` function, but is recognized to provide
      compatibility with the C language `scanf`.- A character that specifies the conversion to be applied.

The exact options that are permitted and how they are interpreted vary
between the different conversion specifiers. See the descriptions of the
individual conversions for information about the particular options that
they allow.
